# Global impact of the first coronavirus disease 2019 (COVID-19) pandemic wave on vascular services

**DOI:** 10.1002/bjs.11961

**Published:** 2020-09-16

**Authors:** The Vascular and Endovascular Research Network (VERN) COVER study collaborative, Ruth A Benson, Sandip Nandhra, Joseph Shalhoub, Nikesh Dattani, Graeme K Ambler, David C Banquet, Ruth A Benson, Sandip Nandhra, Joseph Shalhoub, Graeme K Ambler, Nikesh Dattani, David C Bosanquet, Rachael Forsythe, Sarah Onida, George Dovell, Louise Hitchman, Ryan Preece, Athanasios Saratzis, Chris Imray, Adam Johnson, Andrew Choong, Jun Jie Ng, Sarah Aitken, Jana-Lee Moss, Graeme K Ambler, Ruth A Benson, Sandip Nandhra, Graeme K Ambler, Ryan Preece, Louise Hitchman, Rachael Forsythe, Abhilash Sudarsanam, Adam Tam, Adam W Beck, Adel Barkat, Adnan Bajwa, Ahmed Elbasty, A I Awopetu, Akio Kodama, Aksim G Rivera, Alberto Munoz, Alberto Saltiel, Alejandro Russo, Alex Rolls, Alexandros Kafetzakis, Ali Kimyaghalam, Ali Kordzadeh, Amanda Shepherd, Aminder Singh, Andrea Mingoli, Andreas M Lazaris, Andrej Isaak, Andres Marin, Andrés Reyes Valdivia, Andrew Batchelder, Andrew Duncan, Angeliki Argyriou, Anthony S Jaipersad, Antonio Freyrie, António Pereira-Neves, Anver Mahomed, Arda Isik, Arkadiusz Jawien, Asad J Choudhry, Ashwin Sivaharan, Athanasios Giannoukas, Athanasios Papaioannou, Athanasios Saratzis, Ayman Abbas, Bakoyiannis Christos, Bekir Bogachan Akkaya, Bella Huasen, Bibombe Patrice, Mwipatayi, Bilal Azhar, Boboyor Keldiyorov, Brant W Ullery, Carlo Pratesi, Carlos A Hinojosa, Carlos F Bechara, Carolina Salinas Parra, Charalabopoulos Alexandros, Charlotte Bezard, Cheong Jun Lee, Chris Davies, Christian-Alexander Behrendt, Christopher Lowe, Christos D Karkos, Chun Ling Patricia Yih, Ciarán McDonnell, Claudia Ordonez, Craig Nesbitt, Croo Alexander, Daniel Guglielmone, Daniel T Doherty, David M Riding, Davide Esposito, Denis Harkin, Dennis H Lui, Dhafer M Kamal, Diego Telve, Dimitrios Theodosiou, Domenico Angiletta, Donald Jacobs, Edward Choke, Edward D Gifford, Efthymios Beropoulis, Eftychios Lostoridis, Eleanor Atkins, Elena Giacomelli, Elpiniki Tsolaki, Emma Davies, Emma Scott, Emmanouil Katsogridakis, Ernesto Serrano, Ertekin Utku Unal, Eugenia Lopez, Eustratia Mpaili, Fabrizio Minelli, Fatemeh Malekpour, Fatma Mousa, Felicity Meyer, Felipe Tobar, Filipa Jácome, Flavia Gentile Johansson, Fred Weaver, Gabriel A B Proaño, Gabriel Sidel, Ganesh Kuhan, Gary Lemmon, George A Antoniou, George Papadopoulos, Georgios Pitoulias, Georgopoulos Sotirios, Gerardo Victoria, Gert Frahm-Jensen, Giovanni Tinelli, Giuseppe Asciutto, Gladiol Zenunaj, Gómez Vera Carlos Eduardo, Gonzalo Pullas, Grzegorz Oszkinis, Guriy Popov, Hakkı Zafer İscan, Hannah C Travers, Hashem Barakat, Hayrettin Levent Mavioglu, Ian Chetter, Ian Loftus, Ilias Dodos, Imran Asghar, Isabelle Van Herzeele, Jacopo Giordano, James Cragg, Jason Chuen, Javier Del Castillo Orrego, Jeremy Perkins, João Rocha-Neves, Jorge H Ulloa, José Antonio Chávez, José Vidoedo, Joseph Faraj, Joseph Mills, Juan Varela, Jun Jie Ng, Jürg Schmidli, Kakavia Kiriaki, Katarzyna Powezka, Kathryn Bowser, Katy Darvall, Kenneth McCune, Ketino Pasenidou, Kevin Corless, Kevin McKevitt, Kira Nicole Long, Konstantinos G Moulakakis, Konstantinos Roditis, Konstantinos Stavroulakis, Konstantinos Tigkiropoulos, Kristyn Mannoia, Kumar Abayasekara, Lalithapriya Jayakumar, Lasantha Wijesinghe, Laura Drudi, Lauren Shelmerdine, Leigh Ann O'Banion, Lewis Meecham, Lisa F Bennett, Lorena Grillo, Lucy Green, Lucy Wales, Luís Loureiro, Luis Mariano Palena, Luis Mariano Palena, Mahmoud M H Tolba, Manar Khashram, Manik Chana, Manuel Pabon, Marco González, Marco Virgilio Usai, Marcos Tarazona, Maria A Ruffino, Mariano Castelli, Marie Benezit, Marina Dias-Neto, Martin Malina, Martin Maresch, Martin Mazzurco, Martin Storck, Martín Veras Troncoso, Matt Popplewell, Matteo Tozzi, Matthew Metcalfe, Matti Laine, Mhammed Rawhi, Michael Ricardo, Mingzheng Aaron Goh, Mohamed Abozeid Ahmed, Mohammed Ibrahim, Mohannad Alomari, Muayyad Almudhafer, Muhammed Elhadi, Nalaka Gunawansa, Nancy Hadjievangelou, Natasha Hasemaki, Natasha Shafique, Nathan Aranson, Nicholas Bradley, Nicolas J Mouawad, Nicole C Rich, Nikolaos Floros, Nikolaos Patelis, Nikolaos Saratzis, Nikolaos Tsilimparis, Nilson Salinas, Nishath Altaf, Oliver Friedrich, Oliver Lyons, Olivia M B McBride, Orestis Ioannidis, Orwa Falah, Panagiotis Theodoridis, Paolo Sapienza, Paraskevi Tsiantoula, Patrick Chong, Patrick Coughlin, Paul Bevis, Paul Carrera, Paul Dunlop, Peng Foo Wong, Pereira Albino, Peter Rossi, Petroula Nana, Philip W Stather, Pierfrancesco Lapolla, Pierre Galvagni Silveira, Prakash Saha, Pranav Somaiya, Putera Mas Pian, Rachael L Morley, Rachel Bell, Raed M Ennab, Rafael Malgor, Raffaele Pulli, Ragai Makar, Raghuram, Sekhar, Rana Afifi, Raphael Coscas, Raphael Soler, Robert F Cuff, Rodney Diaz, Rodrigo Biagioni, Rosnelifaizur Bin Ramely, Rubén Rodríguez Carvajal, Sandeep Jhajj, Sara Edeiken, Sergio Benites, Sergio Zacà, Sharath Paravastu, Sharon Chan, Sharvil Sheth, Sherene Shalhub, Shiva Dindyal, Shonda Banegas, Simon Hardy, Simona Sica, Siu Chung Tam, Sivaram Premnath, Sophie Renton, Sriram Rajagopalan, Stavridis Kyriakos, Stavros Kakkos, Stefano Ancetti, Stephane Elkouri, Stephanie Lin, Stephen Wing Keung Cheng, Stylianos G Koutsias, Tabitha Grainger, Tamer Fekry, Tamer Ghatwary Tantawy, Tamim Siddiqui, Taohid Oshodi, Tasleem Akhtar, Thomas James Hardy, Thomas Kotsis, Thushan Gooneratne, Timothy Rowlands, Tina U Cohnert, Tom Wallace, Tristan R A Lane, Umberto Marcello Bracale, Usman Cheema, Uzma Sadia, Vanessa Rubio, Victor Canata, Vincent Jongkind, Vipul Khetarpaul, Virginia Summerour, Walter Dorigo, Wissam Al-jundi, Xun Luo, Yamume Tshomba, Yvis Gadelha Serra

## Abstract

This online structured survey has demonstrated the global impact of the COVID-19 pandemic on vascular services. The majority of centres have documented marked reductions in operating and services provided to vascular patients. In the months during recovery from the resource restrictions imposed during the pandemic peaks, there will be a significant vascular disease burden awaiting surgeons.

**Graphical Abstract:**
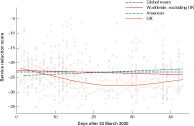

One of the most affected specialties

## Introduction

Coronavirus disease 2019 (COVID-19) has had a profound effect on the availability of surgical resources^1^. Vascular services have been severely affected by these challenges. Some vascular societies have issued guidance on what operative case mix should be undertaken during the pandemic^2–4^. These include adapting service provision for elective and urgent vascular presentations such as stroke and aortic aneurysm. However, the exact impact of the pandemic is still unknown^5^. The Vascular and Endovascular Research Network (VERN) is an established vascular research collaborative^6–9^ that responded rapidly to the pandemic by delivering the COVID-19 Vascular SERvice (COVER) study, an international prospective mixed-methodology project. The aim of the first part of the COVER study described here (tier 1) is to document fluctuations in vascular services globally during the first phase of the pandemic.

**Fig. 1:**
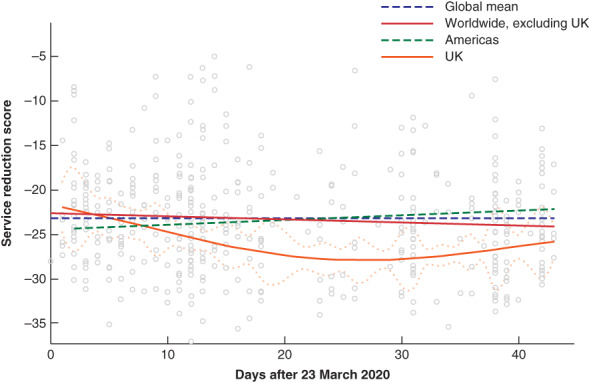
Global overall mean service reductions, worldwide response, and service reduction scores in the UK and the Americas

Grey circles represent centre responses, indicating more populated data at the start and end of the time period. Global overall mean vascular practice was already reduced at the start, remaining relatively steady throughout. Compared with this, vascular surgical units in the UK (lockdown date 23 March 2020) began to implement more significant reductions in services. These measures became steadily more significant until around the time when the pandemic peaked locally (between 10 and 20 April).

## Methods

International guidelines on designing and reporting of surveys were used^10^. The study protocol is available online (https://www.medrxiv.org/content/10.1101/2020.05.27.20114322v1; ISRCTN 80453162).

A remote digital survey was developed by a global team of vascular healthcare professionals. Questions related to all aspects of vascular care, including staff availability, multidisciplinary team input, and personal protective equipment (PPE) (Appendix *S2*, supporting information). Results reported here are for the period 23 March to 3 May 2020, divided into three 2-week periods for comparison. Duplicate responses were removed.

International/continental comparisons were performed, where possible, to describe relative change in practice. A score of 0–3 was allocated to each answer based on perceived relative service reduction by 12 VERN healthcare professionals (0 represents no change and 3 the most significant change) (Appendix *S3*, supporting information).

## Results

Overall, 465 completed survey responses were collected from 249 different units in 53 countries across six continents (*Table S1*, supporting information). *Fig. 1* shows all unit responses together with overall mean service reductions and worldwide response. The reductions in service measures for individual countries are shown in Appendix *S3* (supporting information).

### Carotid surgery

Globally, 17·7 per cent of units offered intervention only to patients with crescendo transient ischaemic attacks, 43·5 per cent continued to offer surgery on a case-by-case basis, and 36·4 per cent made no changes to their carotid practice.

### Aortic screening programmes

Of those units offering aortic aneurysm screening services, 45·8 per cent stopped all screening activities, 18·7 per cent continued a reduced programme, and 7·9 per cent continued screening as usual.

### Aortic pathology

Thresholds for abdominal aortic aneurysm (AAA) repair were raised in the majority of centres; 11·7 per cent of vascular units limited surgery to AAA more than 6·5 cm in maximal diameter, 16·4 per cent to those above 7 cm, 25·1 per cent to symptomatic or ruptured AAA, and 2·3 per cent to AAA suitable for endovascular AAA repair (EVAR) only. Despite this, 25·1 per cent reported no change in practice. Access to EVAR out of hours was initially available to 8·5 per cent of responding units, increasing to 21·2 per cent in the following 4 weeks. Overall, only 14·2 per cent of units maintained a 24/7 EVAR service, 26·3 per cent maintained an ‘in hours’ service, 31·5 per cent offered EVAR for urgent cases only, and 18·5 per cent were able to run their service on an *ad hoc* basis only. Post-EVAR surveillance continued as normal in 24·6 per cent of units. However, 35·2 per cent had reduced availability and 31·8 per cent stopped it completely. The majority of units (56·6 per cent) maintained their pathways for acute aortic syndromes (type B aortic dissection, penetrating aortic ulcer, and intramural haematoma). A small proportion (5·9 per cent) moved to conservative management only, 4·5 per cent were offering early endovascular surgery, and 26·6 per cent limited surgery to ruptures.

### Lower limb

Changes to the management of lower-limb pathology are shown in *Table 1* for each 2-week period. The majority of units began offering a greater proportion of major amputation or palliation rather than attempting revascularization for chronic limb-threatening ischaemia, with 60·4% of units documenting a move to an endovascular-first treatment strategy, especially in critical limbs (rest pain or tissue loss).

**Table 1:** Changes to management of lower-limb arterial pathology during the study period and overall

	Fortnight 1	Fortnight 2	Fortnight 3	Overall
No change	19·7	1·9	13·9	15·4
Increasing endovascular management	19·7	17·2	17·9	18·2
Increasing direct to amputation or palliation	19·7	19·7	25·4	23·6
Limit revascularization to tissue loss	18·9	14·7	15·6	16·3
Limit revascularization to severe rest pain or tissue loss only	19·7	26·7	24·3	23·6
Other	2·4	3·4	2·9	2·9

Values are percentages.

### Outpatient clinics

Some 27·5 per cent of units moved to a triage clinic system, and 29·0 per cent cancelled all planned outpatient clinics. Use of technology permitted 14·9 per cent of units to move to video or telephone clinics, with 18·7 per cent including subsequent triage for attendance if required. The use of ‘hot’ clinics (reserved for acute/urgent patients) increased during the pandemic, and 79·1 per cent of units reported using some form of hot clinic to accommodate vascular patients.

### Multidisciplinary team meetings

Overall, 32·2 per cent of units that normally participated in a multidisciplinary team (MDT) continued with face-to-face meetings; 59·5 per cent stopped regular face-to-face meetings and, of those, 39·1 per cent did not replace them. Overall, 36·8 per cent moved to remote conferencing.

### Staff redeployment

Globally, 5·5 per cent of senior surgeons were redeployed to support other specialties, compared with 53·5 per cent of junior vascular surgical staff.

### Personal protective equipment

The majority (80·5 per cent) of units had PPE guidance in place. Some 26·2 per cent of units did not have access to adequate PPE at the start, compared with 17·5 per cent at the end of the period.

## Discussion

The COVER study is the first international prospective study of unit-level vascular surgical practice during the COVID-19 pandemic. Findings from tier 1 suggest radical changes in practice in a range of services.

One notable change across participating vascular units is the reduction in AAA screening activity. The benefit of AAA screening, and the likelihood of finding a new AAA (less than 1·5 per cent)^11^, must be balanced against the risk of COVID-19 transmission and allocation of resources. Given that the majority of units reported higher size thresholds for AAA intervention, the chances of finding AAAs large enough to be considered for repair at this time are even lower. UK National AAA Screening Programme data suggest that 809 threshold AAAs are identified annually (2018)^12^, which implies that there will be a UK backlog of approximately 130 AAAs relating to this 6-week study period, with resource implications after the pandemic. This will be replicated to some degree worldwide.

Another common finding is the reported preference for endovascular strategies to address aortic and peripheral arterial disease; this is thought to be based on a drive to minimize hospital stay and reduce demand on ICU beds^13,14^. For EVAR, a paradigm has been created where, potentially, more EVAR is performed during the pandemic, but with a reduction in post-EVAR surveillance. There are important implications relating to the financial resources, operating time and staffing that will be required to catch up with missed scans and scheduled operations as services begin to resume. Vascular patients will be competing with the estimated 28 million operations cancelled or postponed during the peak of the pandemic^15^. For lower-limb pathology, the results of an increased endovascular approach on limb-related outcomes will also be important to follow.

MDT meetings support individual clinician decision-making by navigating complex decisions through a multifaceted approach. COVID guidelines^2–4^ have provided recommendations that potentially go against surgeons' usual inclinations. Anecdotally, patients who may have received active treatment before the pandemic were being palliated owing to the perceived high risk of intervention, especially if they tested positive for COVID-19. Strategies have moved towards endovascular management where open surgery would have been the surgeon's usual preference. Replacing a face-to-face MDT with virtual meetings has facilitated ongoing access to MDT support for such complex decision-making during this challenging period.

Despite the large number of units taking part, correlating individual country or regional data with dates of lockdown is challenging. Dates of lockdown were, however, similar for countries providing the majority of responses (UK, Germany, USA). All participating units entered lockdown in March 2020, and were in lockdown when the survey began. If there are any subsequent COVID-19 ‘waves’ in areas that are ‘past the peak’^16–21^, or in locations where the pandemic peak has yet to occur, these data will support vascular surgeons in terms of practice and the resources needed.

## Supplementary Material

bjs11961-sup-0001-Supinfo **Appendix S1**: Supporting information
