# Supplementary material for: Global impact of the first coronavirus disease 2019 (COVID-19) pandemic wave on vascular services
Source: Br J Surg. 2020 Sep 16;107(11):1396–400. doi: 10.1002/bjs.11961 (PMC7929316; doi:10.1002/bjs.11961)
Supplement: bjs11961-sup-0001-Supinfo — Appendix S1: Supporting information [file bjs11961-sup-0001-supinfo.docx]

**BJS_11961**

**Global impact of the first coronavirus disease 2019 (COVID-19) pandemic wave on vascular services**

**The Vascular and Endovascular Research Network (VERN) COVER study collaborative**

# **Appendix S1** List of authors

**Writing committee:**

Ruth A Benson, Sandip Nandhra, Joseph Shalhoub, Nikesh Dattani, Graeme K Ambler, David C Banquet.

**Study Steering committee:**

Ruth A Benson and Sandip Nandhra (study co-leads). Joseph Shalhoub, Graeme K Ambler, Nikesh Dattani, David C Bosanquet, Rachael Forsythe, Sarah Onida, George Dovell, Louise Hitchman, Ryan Preece, Athanasios Saratzis (co-chief investigator), and Chris Imray (co-chief investigator).

**International steering team:**

United States of America: Adam Johnson

Asia (Hong Kong SAR China/Malaysia/Singapore): Andrew Choong, Jun Jie Ng

Australia and New Zealand: Sarah Aitken, Jana-Lee Moss

**Statistical analysis:**

Graeme K Ambler.

**Tier 1 database management and quality assurance:**

Ruth A Benson, Sandip Nandhra, Graeme K Ambler

**Study communications committee:**

Ryan Preece, Louise Hitchman, Rachael Forsythe

**Collaborators:**

Abhilash Sudarsanam, Adam Tam, Adam W. Beck, Adel Barkat, Adnan Bajwa, Ahmed Elbasty, AI Awopetu, Akio Kodama, Aksim G Rivera, Alberto Munoz, Alberto Saltiel, Alejandro Russo, Alex Rolls, Alexandros Kafetzakis, Ali Kimyaghalam, Ali Kordzadeh, Amanda Shepherd, Aminder Singh, Andrea Mingoli, Andreas M. Lazaris, Andrej Isaak, Andres Marin, Andrés Reyes Valdivia, Andrew Batchelder, Andrew Duncan, Angeliki Argyriou, Anthony S Jaipersad, Antonio Freyrie, António Pereira-Neves, Anver Mahomed, Arda Isik, Arkadiusz Jawien, Asad J. Choudhry, Ashwin Sivaharan, Athanasios Giannoukas, Athanasios Papaioannou, Athanasios Saratzis, Ayman Abbas, Bakoyiannis Christos, Bekir Bogachan Akkaya, Bella Huasen, Bibombe Patrice, Mwipatayi, Bilal Azhar, Boboyor Keldiyorov, Brant W. Ullery, Carlo Pratesi, Carlos A. Hinojosa, Carlos F Bechara, Carolina Salinas Parra, Charalabopoulos Alexandros, Charlotte Bezard, Cheong Jun Lee, Chris Davies, Christian-Alexander Behrendt, Christopher Lowe, Christos D. Karkos, Chun Ling Patricia Yih, Ciarán McDonnell, Claudia Ordonez, Craig Nesbitt, Croo Alexander, Daniel Guglielmone, Daniel T Doherty, David M Riding, Davide Esposito, Denis Harkin, Dennis H Lui, Dhafer M Kamal, Diego Telve, Dimitrios Theodosiou, Domenico Angiletta, Donald Jacobs, Edward Choke, Edward D Gifford, Efthymios Beropoulis, Eftychios Lostoridis, Eleanor Atkins, Elena Giacomelli, Elpiniki Tsolaki, Emma Davies, Emma Scott, Emmanouil Katsogridakis, Ernesto Serrano, Ertekin Utku Unal, Eugenia Lopez, Eustratia Mpaili, Fabrizio Minelli, Fatemeh Malekpour, Fatma Mousa, Felicity Meyer, Felipe Tobar, Filipa Jácome, Flavia Gentile Johansson, Fred Weaver, Gabriel AB Proaño, Gabriel Sidel, Ganesh Kuhan, Gary Lemmon, George A Antoniou, George Papadopoulos, Georgios Pitoulias, Georgopoulos Sotirios, Gerardo Victoria, Gert Frahm-Jensen, Giovanni Tinelli, Giuseppe Asciutto, Gladiol Zenunaj, Gómez Vera Carlos Eduardo, Gonzalo Pullas, Grzegorz Oszkinis, Guriy Popov, Hakkı Zafer İscan, Hannah C Travers, Hashem Barakat, Hayrettin Levent Mavioglu, Ian Chetter, Ian Loftus, Ilias Dodos, Imran Asghar, Isabelle Van Herzeele, Jacopo Giordano, James Cragg, Jason Chuen, Javier Del Castillo Orrego, Jeremy Perkins, João Rocha-Neves, Jorge H. Ulloa, José Antonio Chávez, José Vidoedo, Joseph Faraj, Joseph Mills, Juan Varela, Jun Jie Ng, Jürg Schmidli, Kakavia Kiriaki, Katarzyna Powezka, Kathryn Bowser, Katy Darvall, Kenneth McCune, Ketino Pasenidou, Kevin Corless, Kevin McKevitt, Kira Nicole Long, Konstantinos G. Moulakakis, Konstantinos Roditis, Konstantinos Stavroulakis, Konstantinos Tigkiropoulos, Kristyn Mannoia, Kumar Abayasekara, Lalithapriya Jayakumar, Lasantha Wijesinghe, Laura Drudi, Lauren Shelmerdine, Leigh Ann O’Banion, Lewis Meecham, Lisa F Bennett, Lorena Grillo, Lucy Green, Lucy Wales, Luís Loureiro, Luis Mariano Palena, Luis Mariano Palena, Mahmoud MH Tolba, Manar Khashram, Manik Chana, Manuel Pabon, Marco González, Marco Virgilio Usai, Marcos Tarazona, Maria A Ruffino, Mariano Castelli, Marie Benezit, Marina Dias-Neto, Martin Malina, Martin Maresch, Martin Mazzurco, Martin Storck, Martín Veras Troncoso, Matt Popplewell, Matteo Tozzi, Matthew Metcalfe, Matti Laine, Mhammed Rawhi, Michael Ricardo, Mingzheng Aaron Goh, Mohamed Abozeid Ahmed, Mohammed Ibrahim, Mohannad Alomari, Muayyad Almudhafer, Muhammed Elhadi, Nalaka Gunawansa, Nancy Hadjievangelou, Natasha Hasemaki, Natasha Shafique, Nathan Aranson, Nicholas Bradley, Nicolas J Mouawad, Nicole C. Rich, Nikolaos Floros, Nikolaos Patelis, Nikolaos Saratzis, Nikolaos Tsilimparis, Nilson Salinas, Nishath Altaf, Oliver Friedrich, Oliver Lyons, Olivia M.B. McBride, Orestis Ioannidis, Orwa Falah, Panagiotis Theodoridis, Paolo Sapienza, Paraskevi Tsiantoula, Patrick Chong, Patrick Coughlin, Paul Bevis, Paul Carrera, Paul Dunlop, Peng Foo Wong, Pereira Albino, Peter Rossi, Petroula Nana, Philip W Stather, Pierfrancesco Lapolla, Pierre Galvagni Silveira, Prakash Saha, Pranav Somaiya, Putera Mas Pian, Rachael L Morley, Rachel Bell, Raed M Ennab, Rafael Malgor, Raffaele Pulli, Ragai Makar, Raghuram

Sekhar, Rana Afifi, Raphael Coscas, Raphael Soler, Robert F Cuff, Rodney Diaz, Rodrigo Biagioni, Rosnelifaizur Bin Ramely, Rubén Rodríguez Carvajal, Sandeep Jhajj, Sara Edeiken, Sergio Benites, Sergio Zacà, Sharath Paravastu, Sharon Chan, Sharvil Sheth, Sherene Shalhub, Shiva Dindyal, Shonda Banegas, Simon Hardy, Simona Sica, Siu Chung Tam,

Sivaram Premnath, Sophie Renton, Sriram Rajagopalan, Stavridis Kyriakos, Stavros Kakkos, Stefano Ancetti, Stephane Elkouri, Stephanie Lin, Stephen Wing Keung Cheng, Stylianos G. Koutsias, Tabitha Grainger, Tamer Fekry, Tamer Ghatwary Tantawy, Tamim Siddiqui, Taohid Oshodi, Tasleem Akhtar, Thomas James Hardy, Thomas Kotsis, Thushan Gooneratne, Timothy Rowlands, Tina U. Cohnert, Tom Wallace, Tristan R A Lane, Umberto Marcello Bracale, Usman Cheema, Uzma Sadia, Vanessa Rubio, Victor Canata, Vincent Jongkind, Vipul Khetarpaul, Virginia Summerour, Walter Dorigo, Wissam Al-jundi, Xun Luo, Yamume Tshomba, Yvis Gadelha Serra.

**Appendix S2** Tier 1 survey design and methodology

**Tier 1 survey questions:**

"Please tell us your vascular unit/ hospital/institution and city"

"In what country do you work?'"

"Unit / Hospital / Institution"

"State / County (if applicable)"

"City"

"Have you filled this survey in before?"

"Have you modified the working pattern for consultants/attending/faculty "

"within your unit? (clinics / MDT; etc will be covered later in the

survey)"

"Have you or members of your junior team been asked to cross cover other surgical specialties"

"How many vascular Consultants are there in your centre?"

"How many vascular specialist registrars/trainees or equivalent middle grades does your centre have? If outside of the UK - ST refers to year of training e.g. ST3 is someone who is in the 3rd year of training in surgery. PGY: Post graduate year”

"Have you modified the outpatient clinics within your unit?"

"Are you running an emergency or hot clinic for urgent referrals?"

"Are you participating in a face-to-face MDT?"

If you are not running a face-to-face MDT how have you replaced this?"

"Is a vascular scientist/duplex ultrasound service currently available at your centre?"

"What is your centre’s usual primary cross-sectional imaging service?"

"Is your primary cross-sectional imaging service available as normal?"

"If you have an AAA screening programme, is this still running as normal?"

"Do you still have a service running to image patients after an "

"Endovascular aortic repair (EVAR) repair ? (e.g.: annual CT)"

"Is there a full endovascular aortic service available?"

"Are you relying on an increased endovascular strategy service first for Chronic Limb threatening Ischaemia (CLTI)?"

"Do you have vascular specific inpatient beds?"

"Approximately how many vascular specific inpatient beds does your unit "

"normally have?

"How many vascular specific inpatient beds does your unit currently have?"

Have you changed your operative practice for elective AAA surgery?"

"In general have you changed your CLTI revascularisation strategy?"

"In general, have you altered how you manage symptomatic carotid disease?

"In general, have you modified your acute aortic syndrome (type B aortic dissection etc.)”

"Do you have access to a dedicated vascular surgery list daily?"

"If yes - is this running at normal capacity? Any changes to staffing (e.g. theatre team, anaesthetic cover?)"

"If you had access to a hybrid theatre before the pandemic, do you still have normal access to it now?"

"Has your centre disseminated a PPE policy to members of your vascular team / unit?"

"Are you able to follow the policy?"

"Have you got something else to add e.g. a story from your hospital or any comment?"

"If you have completed the survey before, has anything changed at your centre since you last filled it in? "

**Study design and methodology:**

An online survey (SurveyMonkey®) was developed by the COVER study team, with vascular surgeons at junior and senior levels from the USA, UK, Australia and Singapore, to provide clear language and questions that were applicable to global practice assessment.

The survey was piloted amongst the stakeholders in the first instance to ensure language and questions were clear and appropriate.

In the first fortnight, closed and open questions were used enabling each centre to provide free-text for feedback based on the local challenges. The questions related to centres’ provision of common vascular services, imaging, screening, staff availability, theatre suite availability, multi-disciplinary team input, clinics and PPE. After one week, a preliminary review of responses to open questions (marked as ‘other’) was used to provide more closed questions, suitable for the global participants, and to support longitudinal data comparison. Centres were asked, through regular repeated advertisement via social media, e-newsletters, and established international collaborative networks, to complete the survey regularly (at least weekly).

**Data cleaning**

The raw survey data was carefully scrutinised and cleaned prior to analysis. Duplicate responses (defined as responses from the same unit on the same day), and responses which contained no usable data (e.g. where the responder had entered no more than the name and/or size of the unit without answering any of the questions about service provision) were removed. There were a number of responses where the respondent had selected ‘other’ but then typed a free-text response which corresponded with one of the pre-specified options. These options were allocated to specific answers so that they could be counted along with the other options. These were almost exclusively responses made in the first week after the survey went live, before the number of pre-specified options was increased.

**Appendix S3** International/continental comparison analysis

We performed international/continental comparisons, to describe relative change in practice from normal. This was achieved by allocating a score of 0/1/2/3 to each possible answer for each service evaluation question. A score was allocated based on the perceived relative service reduction (with ‘0’ representing no change and ‘3’ representing the most significant change). For example, for the question: “have you changed your operative practice for elective AAA survey?” the answer ‘no change to practice’ automatically scored 0, whereas the answer ‘limiting surgery to >7cm asymptomatic AAA’, a significant change, could be scored 1/2/3. A score for each survey question answer was independently provided by 12 COVER team members (all vascular specialists). The mean value from these responses was then used to quantify the overall change in vascular service provision for each responding unit. Centre responses were then plotted with smoothing splines used to fit the trend in the average response, and Jackknife residuals used to generate approximate 95% confidence intervals for the change in average responses over time (where there was an apparent change in responses over time). Generalised cross-validation was used to automatically choose optimal smoothing parameters.

The scores given to each answer are shown below. ‘*’ indicates responses for which a pre-specified score was mandated.

AAA = abdominal aortic aneurysm, CCU = coronary care unit, CLTI = chronic limb threatening ischemia, HDU = high dependency unit, ITU = intensive care unit, MDT = multidisciplinary team, ST.DEV = standard deviation, TEVAR = thoracic endovascular aortic repair, TIA = transient ischemic attack.

Table A1: VERN Executive Committee member average scores (n=12) for COVER Tier 1 question responses when asked about the perceived significance of each response in terms of service reduction/change:

| QUESTION & HEADLINE RESPONSE | SPECIFIC RESPONSE | MEAN | ST.DEV |
| --- | --- | --- | --- |
| **Have you modified the working pattern for consultants/attending/faculty within your unit?** |  |  |  |
| YES | Consultants asked to support other specialties | 2.58 | 0.51 |
|  | Reduced elective activity | 1.75 | 0.84 |
|  | Urgent intervention only | 2.25 | 0.62 |
|  | Doubled up on call / shadow on call | 1.54 | 0.71 |
|  | Reduced number of consultants in hospital | 1.38 | 0.71 |
| NO* |  | 0.00 | 0.00 |
|  |  |  |  |
| **Have you or members of your junior team been asked to cross cover other surgical specialties** |  |  |  |
| YES | Yes, to medical specialties | 2.42 | 0.80 |
|  | Yes, to the emergency department | 2.25 | 0.75 |
|  | Yes, to ITU / HDU | 2.17 | 0.72 |
|  | Yes, to surgical specialties | 1.17 | 0.39 |
| NO* |  | 0.00 | 0.00 |
|  |  |  |  |
| **Have you modified the outpatient clinics within your unit?** |  |  |  |
| YES | Video/Telephone consultation for all | 1.83 | 0.58 |
|  | All cancelled | 3.00 | 0.00 |
|  | Video/Telephone consultation for triaged patients only | 2.21 | 0.58 |
|  | Triage of referrals and then review those eligible in person | 1.33 | 0.49 |
| NO* |  | 0.00 | 0.00 |
|  |  |  |  |
| **Are you running an emergency or ‘hot’ clinic for urgent referrals?** |  |  |  |
| YES | Yes, 1 - 2 days per week | 1.36 | 0.79 |
|  | Yes, 3 - 4 days per week | 1.64 | 0.67 |
|  | ≥5 per week | 2.00 | 1.26 |
| NO* |  | 0.00 | 0.00 |
|  |  |  |  |
| **Are you participating in a face-to-face MDT?** |  |  |  |
| YES* |  | 0.00 | 0.00 |
| NO* |  | 1.00 | 0.00 |
| ***If you are not running a face-to-face MDT how have you replaced this?*** |  |  |  |
|  | Not being replaced* | 3.00 | 0.00 |
|  | Video conference / teleconference | 1.55 | 0.52 |
|  | Limited Core team attending, others dialling in as required | 1.55 | 0.69 |
|  |  |  |  |
| **Is your primary cross-sectional imaging service available as normal?** |  |  |  |
| YES* |  | 0.00 | 0.00 |
| NO |  | 2.58 | 0.65 |
|  |  |  |  |
| **If you have a AAA screening programme, is this still running as normal?** |  |  |  |
| YES* |  | 0.00 | 0.00 |
| NO | No, Stopped | 2.67 | 0.49 |
|  | No, Reduced | 1.58 | 0.51 |
| (Do not have a screening programme*) |  | 0.00 | 0.00 |
|  |  |  |  |
| **Do you still have a service running to image patients after an EVAR?** |  |  |  |
| YES* |  | 0.00 | 0.00 |
| NO | No, Stopped | 2.42 | 0.51 |
|  | No, reduced | 1.33 | 0.49 |
|  |  |  |  |
| **Is there a full endovascular aortic service available?** |  |  |  |
| YES | Running as normal in hours | 1.18 | 0.82 |
|  | Running as normal 24hours/day* | 0.00 | 0.00 |
|  | Yes but Ad hoc | 1.63 | 0.54 |
|  | Reduced due to COVID related sickness/absence of staff | 2.33 | 0.66 |
| NO | No (only for urgent cases) | 2.54 | 0.47 |
|  |  |  |  |
| **Are you relying on an increased endovascular strategy service first for CLTI?** |  |  |  |
| YES* |  | 1.00 | 0.00 |
| NO* |  | 0.00 | 0.00 |
|  |  |  |  |
| **Do you have vascular specific inpatient beds?** |  |  |  |
| YES* |  | 0.00 | 0.00 |
| NO* |  | 1.00 | 0.00 |
| ***% fall in inpatient vascular beds (compared to normal)*** |  |  |  |
|  | 0 - 25% | 0.96 | 0.85 |
|  | 26 - 50% | 1.83 | 0.61 |
|  | 51 - 75% | 2.46 | 0.50 |
|  | 76 - 100% | 2.75 | 0.62 |
|  |  |  |  |
| **Have you changed your operative practice for elective AAA surgery?** |  |  |  |
| YES | Symptomatic/ruptured only | 2.92 | 0.29 |
|  | Yes, >6.5cm Asymptomatic | 2.04 | 0.62 |
|  | >7cm Asymptomatic | 2.59 | 0.66 |
|  | EVAR only (i.e. only EVAR if anatomically suitable) | 2.42 | 0.64 |
| NO* |  | 0.00 | 0.00 |
|  |  |  |  |
| **In general, have you changed your CLTI revascularisation strategy?** |  |  |  |
| YES | Tissue loss only | 2.50 | 0.80 |
|  | Rest pain or worse | 1.92 | 0.74 |
|  | Increased endovascular strategy | 1.79 | 0.75 |
|  | More conservative decision making | 2.08 | 0.79 |
| NO* |  | 0.00 | 0.00 |
|  |  |  |  |
| **In general, have you altered how you manage symptomatic carotid disease?** |  |  |  |
| YES | Crescendo TIA only | 2.58 | 0.51 |
|  | Case-by-case | 1.71 | 0.81 |
| NO (still following national/international guidelines) * |  | 0.00 | 0.00 |
|  |  |  |  |
| **In general, have you modified your acute aortic syndrome pathway (type B aortic dissection etc.)?** |  |  |  |
| YES | Conservative management, unless ruptured | 2.00 | 0.67 |
|  | Conservative management in a non-ITU/CCU bed | 2.42 | 0.64 |
|  | Early TEVAR and discharge | 1.96 | 0.92 |
| NO* |  | 0.00 | 0.00 |
|  |  |  |  |
| **Do you have access to a dedicated vascular surgery list daily?** |  |  |  |
| YES | Elective vascular lists* | 0.00 | 0.00 |
|  | Reduced but still some during the week | 1.50 | 0.50 |
|  | Urgent theatre cases only | 2.75 | 0.45 |
| NO vascular specific slots/lists |  | 2.42 | 0.50 |
| ***If YES - is this running at normal capacity? Any changes to staffing (e.g. theatre team, anaesthetic cover)?*** |  |  |  |
| Running at normal capacity/no changes* |  | 0.00 | 0.00 |
| Any change specified in free text response* |  | 1.00 | 0.00 |
|  |  |  |  |
| **If you had access to a hybrid theatre before the pandemic, do you still have normal access to it now?** |  |  |  |
| YES | Normal access* | 0.00 | 0.00 |
|  | Reduced access | 1.83 | 0.58 |
| NO | No access at all | 2.83 | 0.39 |

**Reduction to services scoring data:**

Table A2 details the countries that provided more than 10 responses over the survey period and their mean reductions to service (total reduction Mean 23.3, max 37.2, theoretical maximum 47.2)

| **Country Name** | **Continent** | **Responses** | **Units** | **Mean** | **Std.Dev** |
| --- | --- | --- | --- | --- | --- |
| Germany | Europe | 16 | 12 | -17.432 | 5.838 |
| Colombia | Americas | 18 | 14 | -24.698 | 3.736 |
| Italy | Europe | 28 | 15 | -21.707 | 4.474 |
| Greece | Europe | 39 | 17 | -24.008 | 4.057 |
| United States | Americas | 55 | 35 | -23.526 | 4.246 |
| United Kingdom | Europe | 161 | 54 | -25.222 | 5.835 |

**Table S1** Full list of countries surveyed and number of responses

| **Country name** | **Continent** | **Responses** |
| --- | --- | --- |
| United Kingdom | Europe | 161 |
| United States | Americas | 55 |
| Greece | Europe | 39 |
| Italy | Europe | 28 |
| Colombia | Americas | 18 |
| Germany | Europe | 16 |
| Ireland | Europe | 9 |
| France | Europe | 9 |
| Portugal | Europe | 8 |
| Ecuador | Americas | 8 |
| Australia | Oceania | 8 |
| Turkey | Asia | 6 |
| Mexico | Americas | 6 |
| Malaysia | Asia | 6 |
| Argentina | Americas | 6 |
| Spain | Europe | 5 |
| Canada | Americas | 5 |
| Libya | Africa | 4 |
| India | Asia | 4 |
| Hong Kong SAR China | Asia | 4 |
| Brazil | Americas | 4 |
| Bahrain | Asia | 4 |
| Sri Lanka | Asia | 3 |
| Poland | Europe | 3 |
| Israel | Asia | 3 |
| Egypt | Africa | 3 |
| Austria | Europe | 3 |
| Switzerland | Europe | 2 |
| Singapore | Asia | 2 |
| Saudi Arabia | Asia | 2 |
| Russia | Europe | 2 |
| Panama | Americas | 2 |
| New Zealand | Oceania | 2 |
| Netherlands | Europe | 2 |
| Dominican Republic | Americas | 2 |
| Costa Rica | Americas | 2 |
| Bulgaria | Europe | 2 |
| Belgium | Europe | 2 |
| Uzbekistan | Asia | 1 |
| Uruguay | Americas | 1 |
| United Arab Emirates | Asia | 1 |
| Sweden | Europe | 1 |
| Peru | Americas | 1 |
| Paraguay | Americas | 1 |
| Luxembourg | Europe | 1 |
| Jordan | Asia | 1 |
| Japan | Asia | 1 |
| Iraq | Asia | 1 |
| Honduras | Americas | 1 |
| Guatemala | Americas | 1 |
| Finland | Europe | 1 |
| Brunei | Asia | 1 |
| Antigua & Barbuda | Americas | 1 |
